# Temperature-Dependent Nanofabrication on Silicon by Friction-Induced Selective Etching

**DOI:** 10.1186/s11671-016-1438-1

**Published:** 2016-04-27

**Authors:** Chenning Jin, Bingjun Yu, Chen Xiao, Lei Chen, Linmao Qian

**Affiliations:** Tribology Research Institute, Key Laboratory of Advanced Technologies of Materials (Ministry of Education), Southwest Jiaotong University, Chengdu, 610031 Sichuan Province People’s Republic of China; Department of Electronic and Electrical Engineering, University College London, Torrington Place, London, WC1E 7JE UK

**Keywords:** Nanofabrication, Friction-induced selective etching, Etching rate, Temperature, Silicon, AFM

## Abstract

Friction-induced selective etching provides a convenient and practical way for fabricating protrusive nanostructures. A further understanding of this method is very important for establishing a controllable nanofabrication process. In this study, the effect of etching temperature on the formation of protrusive hillocks and surface properties of the etched silicon surface was investigated. It is found that the height of the hillock produced by selective etching increases with the etching temperature before the collapse of the hillock. The temperature-dependent selective etching rate can be fitted well by the Arrhenius equation. The etching at higher temperature can cause rougher silicon surface with a little lower elastic modulus and hardness. The contact angle of the etched silicon surface decreases with the etching temperature. It is also noted that no obvious contamination can be detected on silicon surface after etching at different temperatures. As a result, the optimized condition for the selective etching was addressed. The present study provides a new insight into the control and application of friction-induced selective nanofabrication.

## Background

Owing to its excellent physical and mechanical properties and low cost to obtain, monocrystalline silicon has become a preferred semiconductor material [1]. Silicon is now widely used for micro/nanoelectromechanical systems (MEMS/NEMS) [2], solar photovoltaic battery [3], template for nanoimprint [4], substrate for quantum dot growth and so on [5, 6]. Nanofabrication on silicon is essential to support these applications. At present, plenty of micro/nano-manufacture technologies have been used, including photolithography, nanoimprint lithography, electron beam lithography, probe-based anodic oxidation technology and so on [7]. However, with the requirement for high resolution and low cost, the existing techniques encounter challenges and none of them can satisfy all the needs at the same time [8]. How to fabricate silicon with low cost and high resolution is of much concern.

The probe-based technique enables both site-controlled fabrication and high-resolution measurement of nanostructure dimensions. With the advantages of simple processing, high flexibility, and minor cost, friction-induced nanofabrication provides a convenient method for producing nanostructures on silicon, quartz, glass, and GaAs surfaces more easily than photolithography with etching masks [9–15]. Yu et al. [9] proposed the friction-induced method to fabricate convex nanostructure on silicon surface with a diamond tip. Based on this, Guo et al. [10, 11] produced a series of protrusive hillocks with the height of hundreds of nanometers on silicon surface by the friction-induced selective etching. Furthermore, tribochemistry-induced selective etching can also produce defect-free nanostructures on silicon [12, 14]. The friction-induced selective etching method opens up ways for producing templates for nanoimprint lithography on silicon and quartz, and is expected to fabricate patterned nanochannels in micro/nano fluidic systems for drug delivery, ion transporters, DNA translocators and so on [12, 16]. It also provides a way to modify surface hydrophobicity or tribology properties.

However, it is far from full understanding of the friction-induced selective etching fabrication, and it is of importance to establish a controllable friction-induced selective etching process. It is known that the temperature has a great influence on the chemical reaction, but the effect of the temperature on the friction-induced selective etching remains unknown. Therefore, the present study will be focused on the temperature-dependent etching.

In this study, the effect of etching temperature on the height of the nanostructures, surface roughness and wetting performance was investigated. The hardness and elastic modules of the etched surface were studied by a nanomechanical test system. The chemical composition was studied by an X-ray photoelectron spectroscope (XPS). Based on above investigations, the rate for selective etching and condition for optimized fabrication was further addressed.

## Methods

### Materials

The Si(100) wafers, n-doped with boron, were purchased from MEMC Electronic Materials, Inc., USA. The surface root-mean-square (RMS) roughness was measured as about 0.10 nm over a 2 × 2 μm^2^ area by using an atomic force microscope (AFM; SPI3800N, Seiko Instruments Inc., Tokyo, Japan). Before fabricating, Si wafers were ultrasonically cleaned with acetone, ethanol, and deionized water for 3 min in turn to remove surface contamination. Then, the wafers were dipped in 5 wt.% HF solution for 2 min to etch away the native Si oxide layer, which can facilitate the direct etching of the Si substrate in the following etch process.

### Fabrication

The fabrication process contains two steps, namely scratching and post-etching [11, 12]. With a 5-μm radius diamond tip on the homemade multi-probe instrument [17], a series of grooves with the depth of about 7 nm were produced on a silicon surface. The applied normal load used for the scratching was 20 mN. Then, the samples were etched in 20 wt.% KOH aqueous solution at different temperatures of 0, 25, 40, 50, 60, and 80 °C. To improve the etching quality, isopropanol alcohol (IPA) was added to the KOH solution with the volume ratio of 1:5 [12]. The temperature was controlled via a thermostatic water bath with the accuracy of ±0.5 °C. After the post-etching, the fabricated nanostructures on Si were detected by AFM scanning with a Si_3_N_4_ tip (MLCT, Veeco Instruments Inc., Plainview, NY, USA) in vacuum. The nominal tip radius of the Si_3_N_4_ tip is 20 nm.

### Characterization

To investigate the effect of etching temperature on the performance of silicon surface, a series of detections were conducted on unscratched silicon surface before and after etching at different temperatures. The mechanical properties including hardness and elastic modulus of the etched Si surface were investigated by the in situ nanomechanical test system (TI900, Hysitron Inc., USA). A Berkovich tip was used for the test of hardness *H* and elastic modulus *E*. The indentation test was conducted under a depth-controlled mode. To obtain reliable data, at least three repetitive tests were performed under each indentation depth. The nano-indentation elastic modulus was obtained by analyzing the indentation force-depth (*F-d*) curves with the Oliver-Pharr method [18, 19]. The test was calibrated by a fused quartz wafer with *E* = 69.6 GPa ± 5 % and *H* = 9.25 GPa ± 10 % (Hysitron Inc., USA). The chemical composition of the etched silicon surface was detected by an X-ray photoelectron spectroscopy (XPS; Escalab 250xi, Thermo Fisher Scientific Inc., USA). The contact angle vs. deionized water of the etched Si surface was measured by a drop analysis system (DSA, Kruss Corp., Germany).

## Results and Discussions

### Effect of Etching Temperature on Height of Nanostructures

After immersing the Si surface with groove-shaped scratches in KOH-IPA solution, the protrusive nanostructures were piled up from the scratches. The formation of the protrusive nanostructures (hillocks) was ascribed to the difference in etching rate of the scratched area and original silicon surface, where the scratched area acted as a resist mask against etching [11, 12]. It is also noted that the native oxide layer-covered Si surface can delay the selective etching, where the etching started at about 30 min in 20 wt.% KOH solution [11]. In this study, the native oxide layer was removed by HF solution before consequent etching in KOH-based solution, and hence, obvious selective etching began within much less immersing time.

**Fig. 1:**
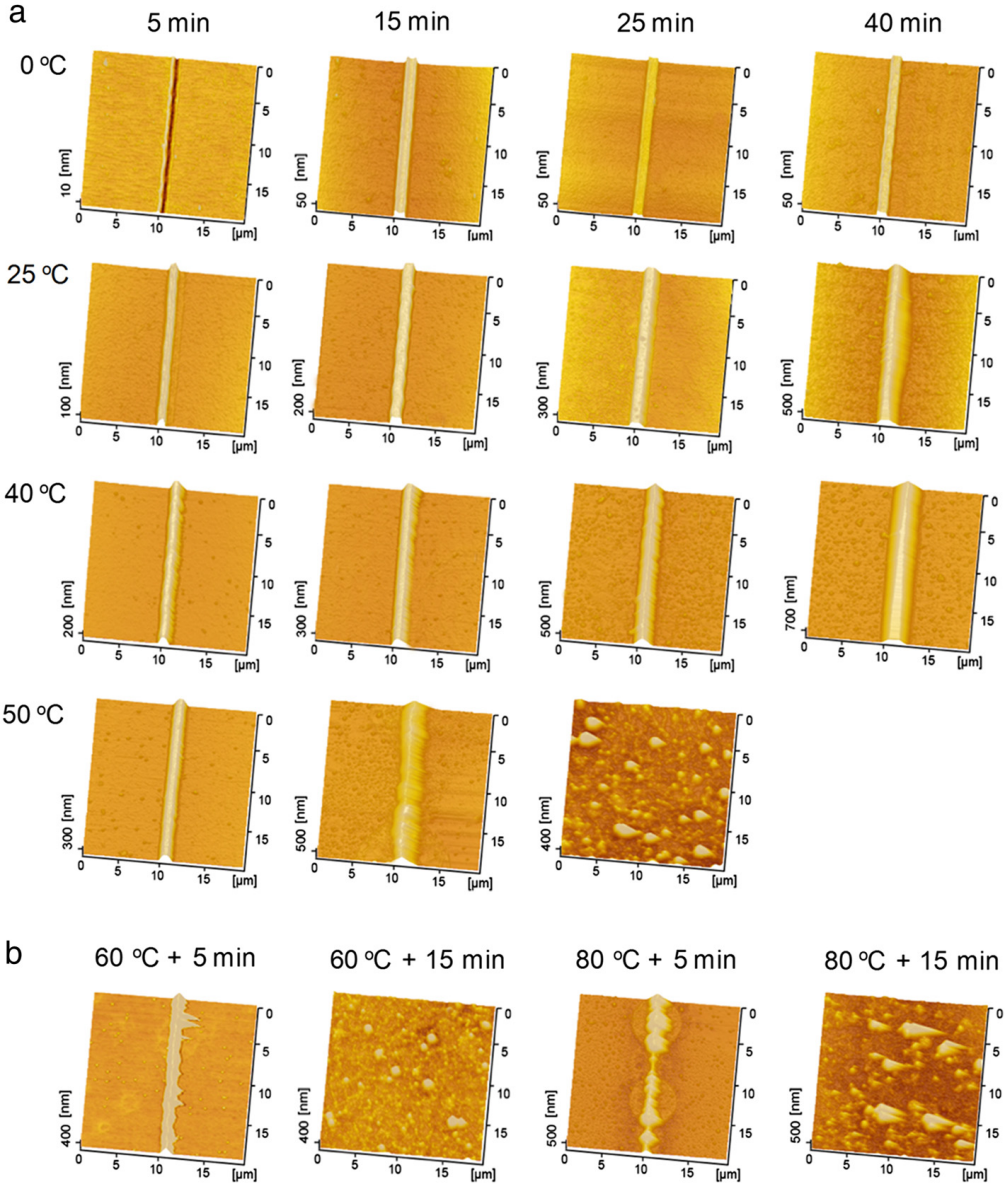
AFM images of the hillocks produced by friction-induced etching under different temperatures. **a** Images of hillocks produced from the scratched area at 0, 25, 40, and 50 °C with the etching time of 5 to 40 min. The surface after etching at 50 °C for 40 min is too rough to be scanned by AFM. **b** Images of the scratched lines after etching at 60 and 80 °C with the etching time from 5 to 15 min. After etching at 50 °C for 25 min, the hillock is completely collapsed, and no obvious pileup can be detected from the scratched area (Fig. 1a). Similar results have been found for 15-min etching at 60 and 80 °C (Fig. 1b)

The formation of the friction-induced hillocks is found to be strongly temperature-dependent. As shown in Fig. 1, after immersing the Si sample in KOH-IPA solution, the protrusive nanostructures piled up from the groove at different etching times and temperatures. The height of the hillock generated by post-etching increased with the etching time slowly at low temperature but sharply at high temperature. It is noted the etching at the temperatures ranging from 0 to 40 °C can facilitate the formation of nice hillocks within 5 to 40 min. However, when the scratched surface was etched at 50 °C, the surface will be severely destroyed after 25 min. With the further increase in the etching temperature, hillocks were hardly produced on the scratched area with the etching time more than 5 min. As shown in Fig. 1b, at 60 °C, no obvious hillock was created from the scratched surface after etching for 15 min. At 80 °C, the selective etching caused easily the collapse of hillocks, and no hillock was detected if the etching time was longer than 5 min.

**Fig. 2:**
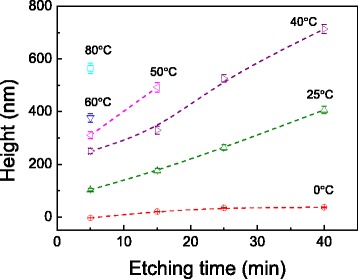
The variation of the hillock height with the etching time after etching under different temperatures

The variation of the hillock height is plotted as a function of etching time under different temperatures, as shown in Fig. 2. From 0 to 40 °C, the height increases with the etching temperature, and higher temperature can lead to a sharper increase of the height with the etching time. At 0 °C, the height increment is 3.2 nm (the difference in the groove depth) after etching for 5 min and 37.0 nm after 40 min. The increment from 5- to 40-min etching is 302.7 nm at 25 °C and 463.9 nm at 40 °C, respectively. In contrast, for higher temperature-etching, a short time (such as 5 min) is enough for hillock formation, while long etching time will lead to the formation of irregular hillocks or damage of surface because of fierce chemical reaction. During etching at high temperature, the deformation layer beneath the scratched area will be etched away rapidly, and then the selective etching will disappear. Long-time etching can also cause the etch-off of the deformation layer even at relative low temperature. Therefore, the hillock will be completely collapsed at high temperature or for a long time. For example, no obvious pileup can be detected from the scratched area after etching at 50 °C for 25 min or at 60 °C for 15 min (Fig. 1). As a result, the etching at temperature from 0 to 40 °C is better for controllable fabrication of hillock.

### Temperature-Dependent Selective Etching Rate

During the friction-induced selective etching process, the chemical reaction of Si and H_2_O takes place in the catalysis of hydroxyl ion OH^−^, and Si(OH)_4_ and H_2_ will be produced as the products [20]. The pre-scratching on silicon can lead to the deformation of silicon crystal matrix, and this will result in a slower etching rate than that on original silicon surface [11]. However, mask effect will disappear when the Si surface is etched for a long time or at a high temperature as shown in Fig. 1. Therefore, we will discuss here the temperature-dependent selective etching based on results from short-time etching up to 40 min.

**Fig. 3:**
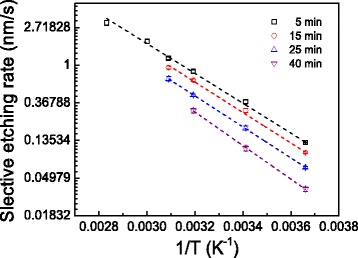
The variation of the selective etching rate with reciprocal temperature on the Si surface. The selective etching height is drawn from the results in Fig. 2, and selective etching rate is obtained by dividing the height difference with etching time. The rate can only be obtained when nice hillock can be produced. The activation energy *E*_a_ can be obtained by measuring the slope based on the Arrhenius equation ln*v*_s_ = ln*A*(c)^*α*^ − *E*_a_/(RT), where *v*_s_ is etching rate, *A* is frequency factor, *c* is reactant concentration, *α* is the reaction order, *R* is gas constant, and *T* is thermodynamic etching temperature, respectively [20]

The selective etching rate was plotted as a function of temperature in Fig. 3. It is noted that the natural logarithm of selective etching rate changes linearly with the reciprocal temperature. According to the Arrhenius equation, the activation energy *E*_a_ for the selective etching can be estimated as 0.33–0.38 eV in the present study. During the etching of silicon by KOH solution, silicon oxide film on silicon can resist the etching of KOH solution as a mask, and cause a slow etching rate at the beginning [10]. In this study, the native oxide layer on silicon was removed by HF solution before fabrication, and hence, it will lead to a rapid etching of silicon compared to the etching of bulk silicon surface [20, 21].

### Effect of Etching Temperature on Surface Roughness

**Fig. 4:**
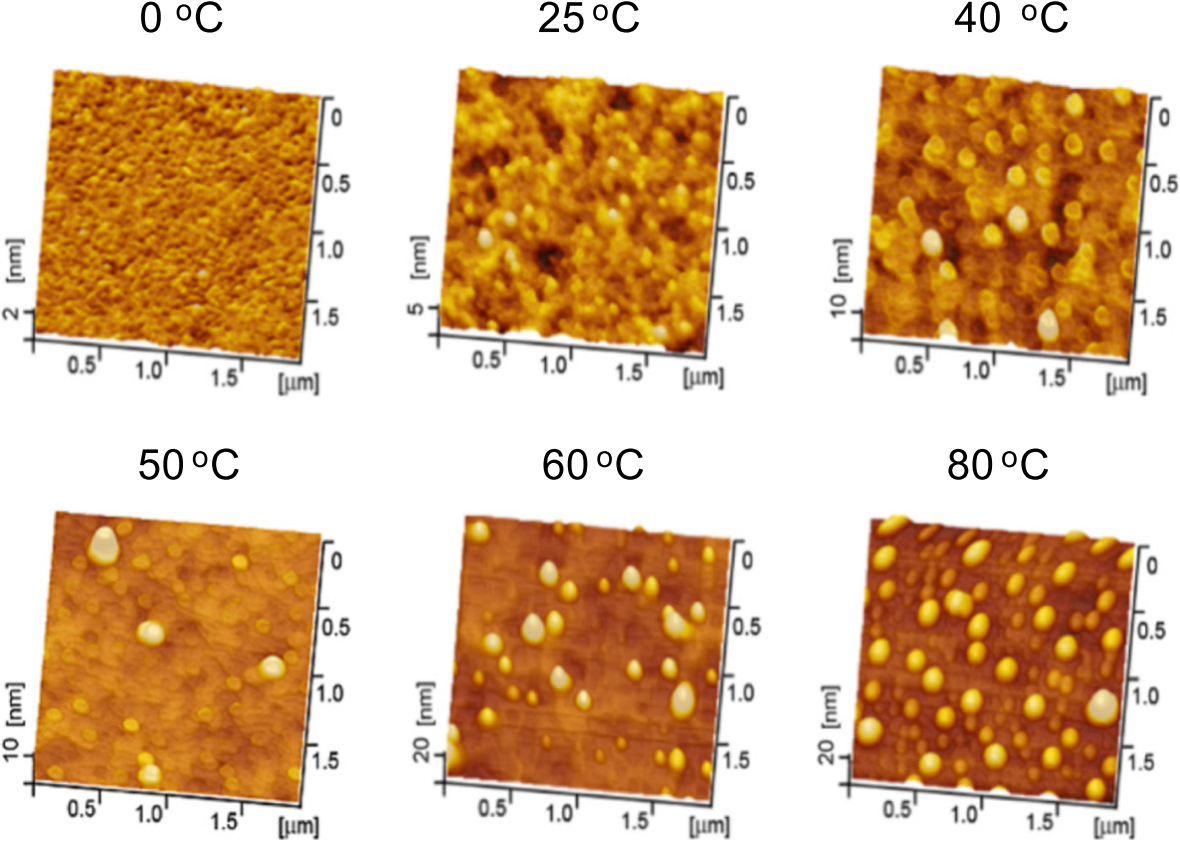
AFM images of Si surface after etching in KOH-IPA solution for 5 min under various etching temperatures

**Fig. 5:**
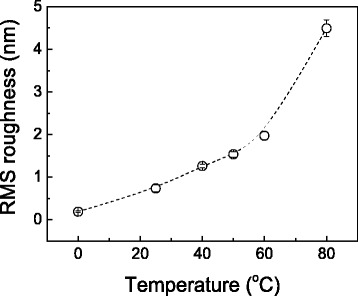
Surface roughness of Si plotted as a function of etching temperature. The etching time was 5 min

In addition to the height of the protrusive nanostructure, the etching temperature also reveals a great influence on the roughness of Si surface. Since the etching time of more than 5 min can lead to the collapse of hillock at high temperature, the etching time of 5 min will be used in the following comparative investigation. Figure 4 illustrates the Si surface etched under various temperatures ranging from 0 to 80 °C, and the corresponding RMS roughness is plotted in Fig. 5. When Si sample is etched at 0 °C, surface RMS roughness is 0.18 nm, which is comparable to the roughness on original silicon surface of 0.10 nm. When the etching temperature is increased from 25 to 60 °C, the roughness increases gradually from 0.76 to 2.0 nm. Nevertheless, severe chemical reactions at high temperature make the Si surface much rougher, and the roughness reaches about 4.6 nm at 80 °C. Meanwhile, the etching for longer time at the same temperature will also make the surface rougher (Fig. 1). It is noted the etching has caused some protrusive spots on the silicon surface especially at high temperature (Fig. 4) or for a long time (Fig. 1), which contributes greatly to surface roughness. During the chemical etching, H_2_ will be produced as a product [20], which can be observed in the present study in the form of tiny bubbles. These bubbles are found to be easily gathered and adhered onto silicon surface especially at high temperature. The formation of these adsorbed bubbles can act as a protection cap to hinder the attack of reactant molecules to the Si surface, and eventually leads to the formation of many protrusive spots [22].

**Fig. 6:**
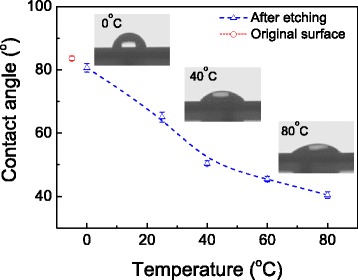
The variation of the contact angle on the Si surface after etching for 5 min at different temperatures. The *inset pictures* show the images for the measurement of surface contact angles

The variation in surface roughness after etching under different temperatures may lead to the difference in surface wetting. To verify this, the contact angles on the etched surface were measured with a water drop as shown in Fig. 6. It is noted that the contact angles decrease with the etching temperature. The original surface produced by etching in HF solution for 2 min presents a contact angle of 83.6°. When the sample is etched in KOH-IPA solution at 0 °C, the contact angle drops to 81.6°. Then, the contact angle keeps decreasing with the increase in the etching temperature, and reaches 39.6 at 80 °C. Therefore, the etching temperature exerts an important influence on surface wetting, and it leads to a decrease in the surface contact angle vs. water.

### Effect of Etching Temperature on Mechanical Properties

**Fig. 7:**
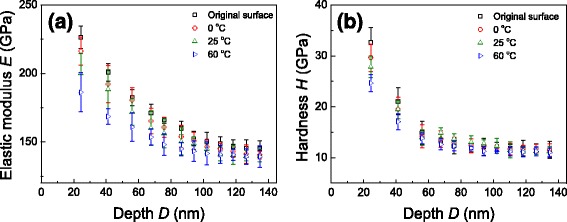
**a** The elastic modulus *E* and **b** hardness *H* measured from the Si surface etched at 0, 25, and 60 °C, respectively. The etching time was 5 min. The measurement on the original surface was plotted as a comparison

The samples were tested by the in situ nanomechanical test system to characterize the micro-mechanical properties. Figure 7 shows the elastic modulus *E* and harness *H* measured from the original surface (etched by HF) and KOH-etched surfaces under three etching temperatures. At the same indentation depth, the elastic modulus of the etched surface is a little lower than that of the original silicon surface (Fig. 7a). It is also noted that etching at higher temperature can lead to a lower elastic modulus than that at a lower temperature. The elastic modulus trends to stabilize at about 150 GPa when the indentation depth exceeds 100 nm. As illustrated in Fig. 7b, the hardness of the etched surface follows a similar trend as the variation of measured elastic modulus. The hardness reaches a stable value of about 11 GPa at the maximum indentation depth higher than 100 nm.

The reason for the temperature-dependent elastic modulus and hardness can be ascribed to the variation in surface rough microstructures. During the etching, surface bonding will be destroyed by the reactants, leaving a rough surface with some sub-structures, such as micro holes, micro walls, and asperities. By contrast to the original surface with compact bonding structure, the etched surface with the sub-structures is expected to degrade the mechanical performance of the Si surface. The etching at a higher temperature can lead to rougher surface with more sub-structures, and hence lower value of elastic modules and hardness [23]. It is also noted that there is an obvious indentation size effect on the tests of elastic modulus *E* and hardness *H*, where the measured value decreases with the indentation depth in the nanoscale. Similar results have been reported on metal or polymer surface [24, 25]. This may be ascribed to the effect of geometrically necessary dislocations in the nanoscale test [24].

### Chemical Composition

**Fig. 8:**
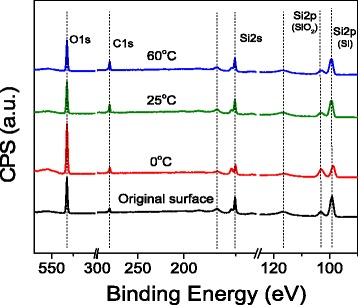
XPS analysis of the etched Si surface after etching for 5 min under different temperatures

To keep the excellent performance of silicon for employment in MEMS/NEMS, no contaminations would be expected in the surface processing [16]. To check the effect of etching on surface composition, XPS detection was performed on silicon surface before and after the etching under various temperatures. As shown in Fig. 8, XPS detection was performed on the original surface and post-etched surface under various etching temperatures (0, 25, and 60 °C), respectively. After etching for 5 min under different temperatures, several peaks are detected from the original surface without etching and from the surface etched at various temperatures. Comparing with the original surface, no extra element peaks or chemical compositions can be found on the etched silicon surface, indicating that a clean surface is produced after etching at different temperatures.

To sum up, the effect of etching temperature on the height of protrusive hillocks, surface roughness of silicon, surface wetting, mechanical performance, and chemical composition was comparatively investigated. Firstly, the removal of surface oxide enables the selective etching, and the hillock height can be controlled by choosing appropriate etching time and temperature as shown in Fig. 2. Secondly, the cooperative effect of etching time and temperature on the fabrication quality should be taken into account for real fabrication. Although high temperature enables a rapid selective etching rate, resulting in high protrusive hillocks, it also causes a rough surface. Considering the collapse of the hillock by excessive etching (Figs. 1 and 2), the temperature below 50 °C is recommended in the present study for selective etching. Since a longer etching time can also cause a rougher silicon surface (Figs. 4 and 5), the etching time should be selected based on the requirement for hillock height and surface roughness. Finally, the selective etching results in a clean surface without contaminations, and the chemical reaction can be predicted by the Arrhenius equation. It should be highlighted that based on the Arrhenius equation, the selective etching process can be further controlled by adjusting the solution concentration, etching temperature, and time. The present study is expected to provide an insight into the understanding, controlling, and application of friction-induced selective nanofabrication.

## Conclusions

The effect of temperature on the friction-induced selective etching was presented for fabricating nanostructures on the Si surface, and the temperature-dependent performance of the silicon surface was addressed. Some main conclusions are summarized as below.

1. The temperature has an obvious effect on the height of the nanostructures and surface roughness produced by friction-induced selective etching. Before the collapse of the hillock, the height of the hillock increases with temperature for the same etching time. The temperature-dependent selective etching process can be fitted well by the Arrhenius equation.
2. Silicon surface becomes rougher with the increasing etching temperature or etching time. The rougher surface produced by selective etching leads to lower contact angles, elastic modulus, and harness than the original surface. No obvious contamination can be detected on the silicon surface after etching at different temperatures
3. Since a high hillock but rough surface can be produced by selective etching at a high temperature or a long time, the temperature below 50 °C is recommended for friction-induced selective etching, and a short etching time can facilitate forming a smooth surface.
